# External validation of a machine learning-based web application for personalized testing of objective functioning using the five-repetition sit-to-stand test

**DOI:** 10.1007/s00701-026-07005-z

**Published:** 2026-08-18

**Authors:** Kenneth Arockia, Massimo Bottini, Anita M. Klukowska, Victor Gabriel El-Hajj, Maria Gharios, Ali Buwaider, Carlo Serra, Luca Regli, Marc L. Schröder, Victor E. Staartjes

**Affiliations:** 1 Machine Intelligence in Clinical Neuroscience & Microsurgical Neuroanatomy (MICN) Laboratory, Department of Neurosurgery, Clinical Neuroscience Center, University Hospital Zurich, University of Zurich, Frauenklinikstrasse 10, 8091 Zurich, Switzerland; 2 Department of Neurosurgery, Medical University of Bialystok, Białystok, Poland; 3 Department of Clinical Neuroscience, Karolinska Institutet, Stockholm, Sweden; 4 Department of Neurosurgery, Medical University of Innsbruck, Innsbruck, Austria; 5 Capio Spine Center Stockholm, Löwenströmska Hospital, Stockholm, Sweden

**Keywords:** Machine learning, Personalized medicine, Precision medicine, Five-repetition sit-to-stand test, Objective functional testing, External validation

## Abstract

**Purpose:**

Simple generalized thresholds for assessing functional impairment in clinical testing are limited, as they fail to consider patient-specific properties such as age, body height, and body mass index. A previously developed machine learning-based model for personalized testing using the five-repetition sit-to-stand (5R-STS) test, that estimates personalized upper limits of normal (ULN) to identify objective functional impairment (OFI) was externally validated to evaluate its performance and generalizability across cohorts.

**Methods:**

Only healthy individuals were included in the study. After the machine learning-based model was applied to this external dataset, expected and observed 5R-STS test times were compared using standardized performance assessment metrics including root mean square error (RMSE), mean absolute error (MAE), and R^2^ values. Additionally, a Bland–Altman analysis was performed to assess agreement between observed and expected values. Validation of the expected ULN involved comparing the proportion of individuals exceeding their personalized thresholds with the corresponding proportion based on the generalized threshold. Subgroup analyses by test setting and country of residence were additionally performed, along with a graphical assessment of model performance.

**Results:**

Application of the model to 171 healthy individuals resulted in an RMSE of 2.33 (95% CI: 1.93 to 2.73) seconds, MAE of 1.70 (95% CI: 1.47 to 1.94) seconds, and R^2^ of 0.064 (95% CI: -0.25 to 0.15). The Bland–Altman analysis demonstrated a mean bias of -1.1 s. Based on the personalized ULNs, OFI was classified in 17.5% of individuals, compared to 6.4% when using the generalized threshold of 10.4 s. These analyses indicated limited external generalization, with acceptable approximation for some faster and mid-range test times but systematic underestimation of slower test times. Multivariable regression analyses showed that remote testing was independently associated with greater prediction bias and higher odds of personalized ULN exceedance compared with supervised testing (adjusted mean difference: − 0.90 s; adjusted OR: 10.7). Exploratory country-of-residence analyses showed differences across the three largest national subgroups. 130 (76.1%) rated ease of use as excellent, and 145 (84.9%) rated clarity of instructions as excellent. 143 participants (83.6%) indicated that they prefer the 5R-STS over a battery of questionnaires.

**Conclusions:**

In the context of personalized testing, moving toward individually focused precision assessment of patients requires rigorous external validation to ensure the robustness of such applied computational methods. In this external validation, the model demonstrated limited generalization including a systematic underestimation of slower test times and insufficient personalized ULN calibration. These findings indicate that external validity of models derived from single-center data can be limited, underscoring the importance of comprehensive external validation and, potentially, multicenter retraining before clinical implementation.

## Introduction

Standardized outcome metrics in neurosurgical practice provide clinicians and researchers with objective measures of a patient’s clinical status and are widely used to evaluate treatment effects and compare outcomes across centers [4, 6, 13, 15, 16]. Traditionally, the thresholds for most such standardized outcome measures have been based on population-derived averages, where the distinction between two categories such as healthy or unhealthy is determined solely based on mean values across a normal population [20].

In the case of the five-repetition sit-to-stand (5R-STS) test, which is used in the diagnostic assessment of degenerative lumbar spine disease, the time required to complete the test is compared with a single upper limit of normal (ULN) derived from a healthy reference cohort. If a patient exceeds that threshold, objective functional impairment (OFI) is classified [8]. In a previous study a population-based ULN of 10.4 s was determined [9]. Although this method provides a straightforward and easily applicable framework, it overlooks certain individual determinants of performance such as age, sex, body weight and body height. For example, taller individuals will inherently have a bias towards longer 5R-STS times as the distance to be covered from a sitting to a standing position is longer [10, 17]. Therefore, this one-size-fits-all approach risks misclassifying patients and limits the precision of standardized outcome assessments.

To address this limitation, a personalized machine learning-based model was developed to improve accuracy in classifying OFI using the 5R-STS test by integrating demographic variables into the estimation of individualized thresholds for test performance, thereby improving precision of OFI classification [17]. While the model demonstrated improved internal performance in patient-specific assessment, its generalizability across different demographics, testing conditions, and calibration settings remain to be determined [17].

External validation remains a crucial step in confirming a model’s predictive power by applying it to contexts and datasets that differ from those used during training [11, 18]. Successful external validation adds proof to the model’s adaptability and accuracy such that it may be considered for integration into clinical and research workflows [2]. In the current era of precision medicine, a strategy’s adaptability to different environments and patient cohorts has become a key determinant of its clinical value [5, 7, 12]. Therefore, in this study the previously published personalized machine learning-based web application for outcome assessment using the 5R-STS test is externally validated on an independent cohort of healthy individuals.

## Materials and methods

### Study cohort

All included individuals were healthy and had no prior diagnosis of functional impairment regarding the spine. The data for external validation was pooled between April 2024 and April 2025 from participants across Switzerland, Sweden, the United States of America, Lebanon, France, Italy, Cyprus, Saudi Arabia, Croatia, Poland, the United Kingdom, the United Arab Emirates, Austria, Canada and Senegal. As the model used in this study is intended to estimate individualized normal 5R-STS test times based on sociodemographic information, the external validation in a cohort of individuals without known impairment is essential to assess calibration, systematic bias, and false-positive classification.

It should be noted that the sample size for this study was determined by the number of eligible healthy participants available during the study period. As recommended for external validation of prediction models, the focus was therefore on the precision of performance estimates rather than hypothesis testing. Accordingly, the results should be interpreted in light of the reported 95% confidence intervals, which reflect the uncertainty associated with the validation sample.

### Ethical approval

An exemption from ethical review was granted by the appropriate review board (Medical Research Ethics Committees United, Registration Numbers: W17.107 and W17.134).

Consent to participate: Healthy volunteers were thus recruited, and informed consent was obtained from each participant before recording data.

The study was conducted in accordance with the Declaration of Helsinki and its amendments.

### Measurement and data collection

The 5R-STS test was performed according to a previously published standardized testing protocol [9]. Specifically, an armless, hard-seated chair of standard height (48 cm) was positioned firmly against a wall, stable shoes were worn, and participants were instructed and motivated to perform the test “as fast as possible”. The 5 repetitions were timed from the “go” command to the completed fifth stand (5R-STS test time). If the participant was unable to perform the test in 30 s, or not at all, this was noted and the test score was recorded as 30 s. Individuals who performed the test remotely followed the identical protocol after having received precise instructions. A previous study has demonstrated that remotely collected 5R-STS test time measurements do not differ significantly from supervised assessments [19]. Therefore, these data were included in the analysis.

Participants also completed questionnaires to provide sociodemographic information, including sex, age, body weight, body height, smoking status, and highest level of education.

### Model description

This study represents an external validation of the previously constructed, trained and internally validated machine learning-based model and web application for personalized quantification of OFI derived from the 5R-STS test (https://neurosurgery.shinyapps.io/5RSTS/) [17]. The original model is a quantile regression model with a least absolute shrinkage and selection operator (Lasso) penalty and was trained for the 2.5th, 50th, 97.5th, and 99th quantiles (tau). This model was trained to predict an expected test time and a personalized ULN based on basic demographic data, which included age, height, body mass index (BMI), gender and smoking status, as these have a known influence on test performance [10]. The personalized ULN is a model-derived prediction for a healthy individual with identical demographic characteristics. Individuals whose observed test times exceed their model-derived ULN are thus classified as objectively functionally impaired. Additionally, a web application was developed to achieve a user-friendly implementation of automatized test time assessment.

### External validation

For external validation, the original model was applied without any modification to an independent dataset. The demographic variables were standardized and pre-processed according to the same rules defined in the original study and similarly the expected test time and personalized ULN were used for further performance assessment of the model. The ease of use and clarity of instructions was also rated by all participants from 0 to 10, with scores of 8 to 10 being regarded as “excellent” ease of use/clarity of instructions. Furthermore, we asked participants whether they would prefer performing the 5R-STS or filling in a battery of questionnaires for their baseline and follow-up assessment.

### Statistical analysis

All columns and variables not included among the regression predictors (age, sex, body height, body weight, smoking status) were excluded prior to analysis. Further, all values were standardized according to the variable definitions used by the model (e.g. smoking status has 3 levels: 1 for “Current”, 2 for “Previous” and 3 for “Never”). The mean of the two reported test times corresponding to the two attempts was used for analysis to reduce measurement variability. Continuous or numerical data was reported as mean ± standard deviation.

The original model was then applied to the preprocessed data to estimate the 2.5th, 50th, 97.5th, and 99th percentile values for each individual. Model performance was assessed by comparing the model’s personalized prediction for the 50th quantile (median) with the corresponding observed test time reported in the dataset. Primary performance metrics including resampled root mean square error (RMSE), mean absolute error (MAE), and coefficient of determination (R^2^), as well as their 95% confidence intervals (CIs) were computed using the bootstrap method with 2000 iterations per metric. Agreement between observed and expected test times was further assessed using a Bland–Altman analysis, which compares the difference and average of two corresponding values to determine systematic bias, limits of agreement and proportional bias.

It should be noted that primary performance metrics were limited to the predicted 50th percentile. As only a single quantile from the entire outcome distribution is assessed, variability outside the central tendency is not captured, which inherently limits the significance and explanation of variance shown in metrics such as R^2^.

A graphical performance assessment was included to visually evaluate model fit and calibration. A scatter plot of expected versus observed 5R-STS test times with a superimposed locally weighted regression (LOESS) smoother and the line of identity (y = x) as well as a QQ-plot comparing expected and observed test times were included.

Subsequently, a ULN validation was conducted, where the proportion of healthy individuals whose observed 5R-STS time was below their personalized ULN (99th percentile) was calculated. The observed coverage was compared with the nominal 99% expectation and with the proportion determined using the population-based ULN of 10.4 s.

Additionally, multivariable regression analyses were performed to assess potential sources of heterogeneity in model performance. Separate models were fitted to evaluate whether test setting, defined as remote versus supervised assessment, and country of residence, restricted to the three largest national subgroups, were independently associated with prediction bias and ULN exceedance rates. Models were adjusted for relevant demographic and anthropometric covariables. These analyses were intended to evaluate whether test setting or country of residence contributed to differences in model calibration and prediction error.

The study was reported according to the TRIPOD checklist for transparent reporting of prediction model validation. Analyses were carried out using R version 4.4.3 (The R Foundation for Statistical Computing, Vienna, Austria) [14].

## Results

### Patient cohort

A detailed report of all relevant characteristics of the involved cohort can be found in Table 1. Specifically, the mean recorded 5R-STS test time across the population was 7.1 ± 2.1 s, the mean age was 33.9 ± 15.1 years, mean height was 173.0 ± 9.8 cm, mean weight was 73.8 ± 14.5, mean BMI was 24.6 ± 3.9 kg/m^2^, 81 individuals were male (47.4%), and the smoking status was distributed as follows: 23 individuals (13.5%) were active smokers, 18 (10.5%) had ceased smoking, and 130 (76.0%) had never smoked.

**Table 1:** Summary of baseline parameters of the 171 healthy individuals included in the external validation cohort, continuous variables are presented as mean ± standard deviation, and categorical variables as absolute numbers and percentages

Parameters of (*n* = 171) participants	
5R-STS test time [sec], mean ± SD	7.1 ± 2.1
Age [yrs.], mean ± SD	33.9 ± 15.1
Male gender, *n* (%)	81 (47.4)
Height [cm], mean ± SD	173.0 ± 9.8
Weight [kg], mean ± SD	73.8 ± 14.5
Body Mass Index [kg/m2], mean ± SD	24.6 ± 3.9
Smoking status, *n* (%)	
Active smoker	23 (13.5)
Ceased smoking	18 (10.5)
Never smoked	130 (76.0)
Highest level of education, *n* (%)	
High school	31 (18.1)
Higher education	121 (70.8)
Doctorate	19 (11.1)
Remote or Supervised test performance, *n* (%)	
Remote	119 (69.6)
Supervised	52 (30.4)
Ease of use rated between r = 0 and r = 10, *n* (%)	
Rating r = 0	1 (0.6)
Rating r = 1	0 (0.0)
Rating r = 2	0 (0.0)
Rating r = 3	2 (1.2)
Rating r = 4	0 (0.0)
Rating r = 5	5 (2.9)
Rating r = 6	2 (1.2)
Rating r = 7	31 (18.1)
Rating r = 8	34 (19.9)
Rating r = 9	22 (12.9)
Rating r = 10	74 (43.3)
Clarity of instructions rated between r = 0 and r = 10, *n* (%)	
Rating r = 0	0 (0.0)
Rating r = 1	0 (0.0)
Rating r = 2	2 (1.2)
Rating r = 3	1 (0.6)
Rating r = 4	3 (1.8)
Rating r = 5	4 (2.3)
Rating r = 6	3 (1.8)
Rating r = 7	13 (7.6)
Rating r = 8	22 (12.9)
Rating r = 9	15 (8.8)
Rating r = 10	108 (63.2)
5R-STS versus questionnaire preference, *n* (%)	
5R-STS	143 (83.6)
Questionnaire	28 (16.4)
Country of residence, *n* (%)	
Sweden	54 (31.6)
Lebanon	45 (26.3)
Poland	26 (15.2)
The United Arab Emirates	15 (8.8)
The United States of America	9 (5.3)
France	6 (3.5)
The United Kingdom	5 (2.9)
Croatia	3 (1.8)
Switzerland	2 (1.2)
Canada	1 (0.6)
Italy	1 (0.6)
Cyprus	1 (0.6)
Saudi Arabia	1 (0.6)
Austria	1 (0.6)
Sénégal	1 (0.6)

### Personalized test time quantiles

**Fig. 1:**
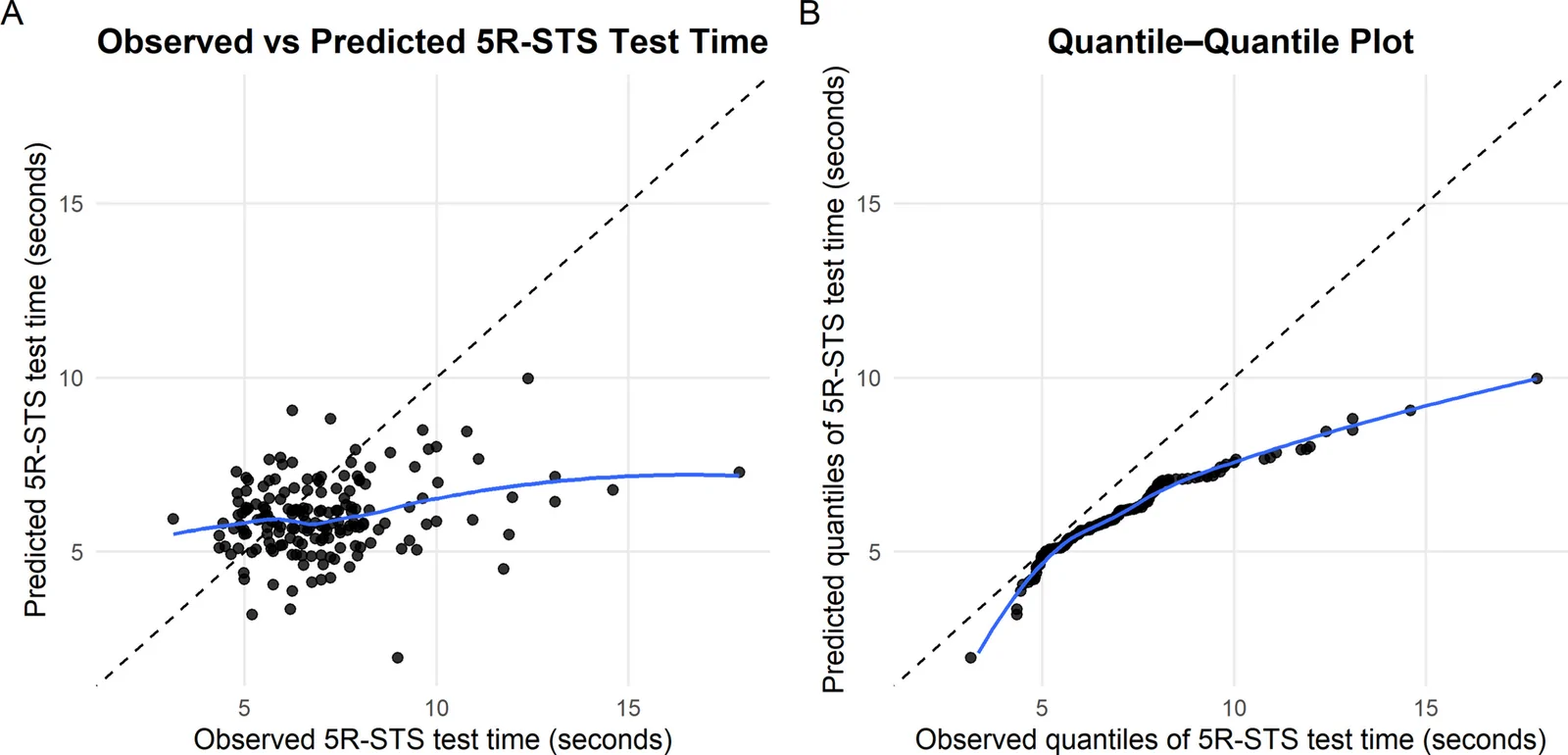
A scatter plot of expected (expected 50% percentile of the model) versus observed 5R-STS test times (Panel A) and a QQ-plot comparing the quantiles of expected (expected 50% percentile of the model) versus observed 5R-STS test times (Panel B) each with a superimposed locally weighted regression (LOESS) smoother and the line of identity (y = x)

**Fig. 2:**
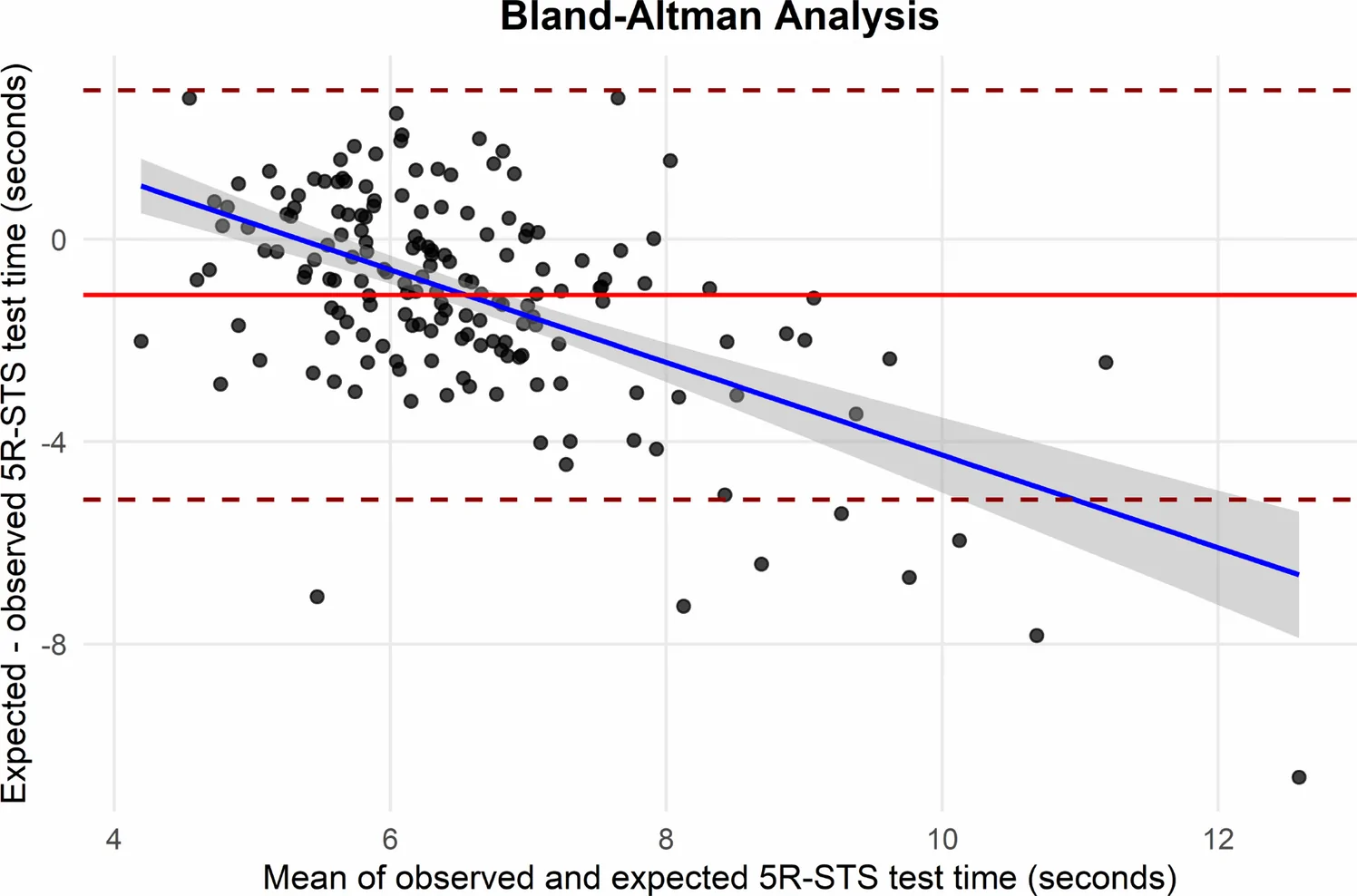
Bland–Altman plot showing the difference between expected and observed 5R-STS test times against their mean. The solid horizontal line indicates the mean bias, dashed horizontal lines indicate the 95% limits of agreement, and the linear regression line illustrates proportional bias. A negative slope indicates increasing underestimation at longer 5R-STS test times

The primary performance metrics demonstrated that the model achieved an RMSE of 2.33 s (95% CI: 1.93 to 2.73), an MAE of 1.70 s (95% CI: 1.47 to 1.94), and R^2^ of 0.064 (95% CI: −0.25 to 0.15). Additionally, the correlation between observed and expected test times was 0.25 (95% CI: 0.11 to 0.39). The Bland–Altman analysis reported a mean bias of −1.1 s, with 95% limits of agreement from −4.6 s to 3.5 s. Formal assessment of proportional bias demonstrated a significant negative association between the mean of observed and expected test times and their difference (slope: −0.92, *p* < 0.001). A summary of the mentioned performance metrics can be found in Table 2. The scatter plot and QQ-plot with LOESS for the graphical performance assessment may be found in Fig. 1. The Bland–Altman plot has been included in Fig. 2.

**Table 2:** Performance metrics of the externally validated model, where reported 5R-STS test times of the participants (N = 171) are compared to the corresponding predictions of the expected median test time (tau = 0.50, 50th percentile). Bland–Altman analysis included assessment of mean bias, 95% limits of agreement, and proportional bias

**Performance Measure**	**External Validation Performance** **(95% CI)**
Root Mean Square Error (RMSE), [sec.]	2.33 (1.93 to 2.73)
Mean Absolute Error (MAE), [sec.]	1.70 (1.47 to 1.94)
R^2^	0.064 (−0.25 to 0.15)
**Bland–Altman Analysis**	**Result**
Mean bias [sec.]	−1.1
95% limits of agreement **[sec.]**	− 4.6 to 3.5
Proportional bias slope β	−0.92; *p* < 0.001

### ULN validation

From the entire cohort, the proportion of individuals with a lower observed 5R-STS test time than their personalized ULN was 82.5% (95% CI: 75.2% to 87.5%), compared to the expected nominal coverage of 99% and the proportion of individuals determined by the population-based ULN of 10.4 s which was computed to be 93.6%.

### Subgroup analysis by test setting and country of residence

In multivariable regression analyses, test setting was independently associated with model calibration. Compared with supervised testing, remote testing was associated with a larger negative bias, indicating greater underestimation of observed 5R-STS test times (adjusted mean difference −0.90 s, 95% CI −1.63 to −0.17; *p* = 0.016). Remote testing was also associated with higher odds of exceeding the personalized ULN (adjusted odds ratio (OR) 10.7, 95% CI 2.28 to 82.9; *p* = 0.007). In the exploratory analysis of the three largest national subgroups, participants residing in Lebanon and Poland showed more negative adjusted bias compared with Sweden, although these differences did not reach statistical significance (Lebanon vs. Sweden: −0.91 s, 95% CI −1.85 to 0.02; *p* = 0.056; Poland vs. Sweden: −1.13 s, 95% CI −2.29 to 0.03; *p* = 0.057). Personalized ULN exceedance was significantly more likely among participants from Lebanon compared with Sweden (adjusted OR 9.73, 95% CI 2.04 to 63.0; *p* = 0.008), whereas the difference between Poland and Sweden was not statistically significant (adjusted OR 2.94, 95% CI 0.40 to 21.0; *p* = 0.269). Descriptive subgroup metrics, including sample size, bias, MAE, RMSE, and ULN exceedance rates, are provided in Table 3.

**Table 3:** Subgroup comparison of model performance, where model performance is shown by test setting and country of residence for the three largest national subgroups. Bias was calculated as expected minus observed 5R-STS time; negative values indicate underestimation of observed performance. MAE, RMSE, and personalized ULN exceedance are reported for each subgroup

Subgroup analysis	Group	n	Bias, [sec.]	MAE, [sec.]	RMSE, [sec.]	Personalized ULN exceedance
Test setting	Supervised	52	−0.57	1.26	1.72	2/52 (3.9%)
	Remote	119	−1.33	1.89	2.56	29/119 (24.4%)
Country of residence	Sweden	54	−0.24	1.18	1.50	3/54 (5.6%)
	Lebanon	45	−1.46	1.95	2.83	11/45 (24.4%)
	Poland	26	−1.96	2.67	3.22	8/26 (30.8%)

### Ease of use, clarity of instructions, and 5R-STS preference

130 (76.1%) of participants rated the ease of use of the web application as excellent, and 145 participants (84.9%) rated the clarity of instructions as excellent (scores ≥ 8). A total of 143 participants (83.6%) indicated that they would prefer performing the 5R-STS to instead filling in a battery of questionnaires.

## Discussion

This study represents the external validation of a machine learning-based model and web application for functional testing using the 5R-STS test. While the previously published study developed and validated the model on an internal cohort, this work evaluates its generalizability across diverse populations. The model was applied to a cohort of 171 healthy individuals to predict personalized 5R-STS test times as well as personalized ULNs for each participant. The evaluation of agreement between reported and model-derived expected test times demonstrated a weak association between the values for faster test times, whereas predictions consistently underestimated slower performance times.

Most clinical testing thresholds distinguishing between healthy and impaired individuals are derived from population-level averages and applied uniformly, without accounting for individual differences in testing characteristics. In the case of the 5R-STS test, a single upper limit of normal (ULN) is commonly used to diagnose objective functional impairment (OFI), regardless of sociodemographic factors. Although separate thresholds could theoretically be calculated for specific subgroups, such an approach would be cumbersome and impractical in clinical use. Machine learning–based models can overcome these limitations by automatically estimating individualized ULNs based on simple sociodemographic inputs. In the case of the model evaluated in this study, internal validity has been assessed previously, however rigorous external validation is required to test the robustness and adaptability of the model to different testing conditions. Ultimately, external validation is a key step toward clinical implementation or regulatory approval of such applied computational methods.

The external assessment conducted in this study showed a measurable but weak association between expected and observed test times, while ULN validation indicated acceptable coverage in some individuals but insufficient overall calibration of the personalized threshold. The performance assessment using RMSE (2.33 s) and MAE (1.70 s) demonstrated non-negligible absolute prediction error. Compared with the mean observed 5R-STS test time of 7.1 ± 2.05 s, however, these deviations represent a relevant proportion of the observed performance range in this external cohort. The low R^2^ value of 0.064 indicates that the model explained only a small proportion of the variance in observed 5R-STS test times. As a personalized model is expected to capture meaningful interindividual differences, this finding suggests limited individual-level predictive value and indicates the need for recalibration or multicenter retraining before clinical implementation.

Further, considering the Bland–Altman analysis, the graphical assessment and the ULN validation a tendency to underestimate slower test times can be observed. This was supported by formal proportional bias testing, which showed a significant negative association between average test time and prediction error (slope: −0.92, *p* < 0.001). Regarding ULN validation, only 82.5% of individuals in the healthy cohort had an observed 5R-STS test time below their personalized ULN, compared with the expected nominal coverage of 99% and 93.6% using the population-based threshold of 10.4 s. This corresponds to a false-positive rate of 17.5%, meaning that nearly one in five healthy individuals would have been incorrectly classified as having OFI based on the personalized threshold. Thus, calibration of the model-derived 99th percentile was insufficient in this external cohort, representing an important clinical limitation for screening purposes. In comparison, the population-based threshold of 10.4 s showed higher specificity in this cohort, with 6.4% of healthy individuals classified as having OFI.

These limitations may be partly explained by the subgroup analyses. In multivariable regression, remote testing was independently associated with greater underestimation than supervised testing (adjusted mean difference in bias: −0.90 s; *p* = 0.016) and higher personalized ULN exceedance (adjusted OR: 10.7; *p* = 0.007), suggesting that unsupervised testing conditions may affect 5R-STS performance and model accuracy. In addition, the exploratory country-level analysis showed more negative adjusted bias in participants residing in Lebanon and Poland compared with Sweden, and significantly higher personalized ULN exceedance in participants residing in Lebanon. Although limited subgroup sizes require cautious interpretation, these findings suggest that test setting and cohort composition should be considered in future validation and recalibration studies.

The mismatch in accuracy regarding slower and faster test times may have occurred due to differences in sociodemographic characteristics between the population the model was trained on and the validation dataset, variations in the protocol for performing the 5R-STS test such as minor differences in chair height or timing methods, or even due to sparse training data for individuals with longer test times.

Nonetheless, it must be stressed that a perfect prediction of 5R-STS test times only based on sociodemographic factors isn’t to be expected at all. Rather, the machine learning-based model and web application are intended to create a personalized, but at the same time easy to use strategy for clinical testing. Especially the web application emphasizes this concept. Also, most participants rated the web application favorably, which supports the initiative to implement automated testing tools into clinical assessment workflows. Even though the model might not perfectly estimate a patient’s test time, by recording simple demographic data using a short questionnaire this model provides the clinician or researcher with a threshold that is tailored and personalized to the patient. This personalized approach remains conceptually attractive and may be considered superior to generalized cutoffs after appropriate recalibration and retraining.

Moving forward, this method of testing shall be refined using more data to train such models and improve their accuracy. Further personalization might be added through machine vision, or motion tracking to more reliably assess the patient’s functional outcomes [1, 3].

## Conclusions

In the context of personalized testing, moving toward individually focused precision assessment of patients requires rigorous external validation to ensure the robustness of such applied computational methods. Although the model provided approximate estimates for some individuals, this study demonstrates that external validity of models derived from single-center data can be limited, underscoring the importance of comprehensive external validation and, potentially, multicenter retraining before clinical implementation. We also demonstrate that personalized testing with this type of web application is seen as easy, and preferable by participants.

## Data Availability

The participant-level data generated and analyzed in this study are not made publicly available, due to data protection and ethical concerns. Certain data may be provided by the corresponding author upon request and appropriate approval.
